# Effects of computer-assisted navigation versus conventional total knee arthroplasty on the levels of inflammation markers: A prospective study

**DOI:** 10.1371/journal.pone.0197097

**Published:** 2018-05-14

**Authors:** Shu-Jui Kuo, Horng-Chaung Hsu, Ching-Jen Wang, Ka-Kit Siu, Ya-Hung Hsu, Jih-Yang Ko, Chih-Hsin Tang

**Affiliations:** 1 Graduate Institute of Clinical Medical Science, China Medical University, Taichung, Taiwan; 2 Department of Orthopedic Surgery, China Medical University Hospital, Taichung, Taiwan; 3 Department of Orthopedic Surgery, Kaohsiung Chang Gung Memorial Hospital, Kaohsiung, Taiwan; 4 Core lab for phenomics and diagnostics, Kaohsiung Chang Gung Memorial Hospital, Kaohsiung, Taiwan; 5 Center for shockwave medicine and tissue engineering, department of medical research, Kaohsiung Chang Gung Memorial Hospital, Kaohsiung, Taiwan; 6 Department of orthopedic surgery, Xiamen Chang Gung Hospital, Fujian, China; 7 Chinese Medicine Research Center, China Medical University, Taichung, Taiwan; 8 Department of Pharmacology, School of Medicine, China Medical University, Taichung, Taiwan; 9 Department of Biotechnology, College of Health Science, Asia University, Taichung, Taiwan; Hospital del Mar, SPAIN

## Abstract

Total knee arthroplasty (TKA) is a well-established modality for the treatment of advanced knee osteoarthritis (OA). However, the detrimental effects of intramedullary reaming used in conventional TKA for distal femur cutting are of concern. Avoiding intramedullary reaming with the use of computer-assisted navigation TKA can not only provide superior prosthetic alignment, but also mitigate perioperative blood loss and the dissipation of marrow emboli. We quantified local and systemic concentrations of inflammation markers for both techniques. Forty-four participants undergoing computer-assisted navigation and 53 receiving conventional TKA for advanced knee OA were recruited between 2013/02/08 and 2015/12/09. Blood samples were collected from all participants at baseline then again at 24 and 72 hours postoperatively and analyzed by ELISA for interleukin 6 (IL-6), IL-10, tumor necrosis factor alpha (TNF-α) and transforming growth factor beta 1 (TGF-β1); these markers were also measured in Hemovac drain fluid collected at 24 and 72 hours. Serum levels of IL-6, IL-10, TNF-α and TGF-β1(unit for all markers: pg/mL) were increased from baseline by smaller increments in the navigation TKA cohort compared with the conventional TKA group at 24 hours (17.06 vs 29.39, p = 0.02; 0.51 vs 0.83, p = 0.16; –0.04 vs 0.36, p < 0.01 and –48.18 vs 63.24, p< 0.01, respectively) and at 72 hours (12.27 vs 16.87, p = 0.01; –0.40 vs 0.48, p < 0.01; 0.58 vs 0.98, p = 0.07 and –55.16 vs 63.71, p < 0.01, respectively). IL-10 levels in drainage fluids collected 24 hours after TKA were also significantly lower in the navigation group versus the conventional TKA group (8.55 vs 12.32, p < 0.01). According to our evidence, the merits of computer-assisted navigation TKA are augmented by low levels of inflammation markers.

## Introduction

Total knee arthroplasty (TKA) is a well-established modality for the treatment of advanced knee osteoarthritis and is associated with high rates of patient satisfaction [1]. However, intramedullary reaming and insertion of distal femur cutting jigs stimulates the dissipation of marrow emboli, which may increase the perioperative risk of acute myocardial infarction (AMI) or cardiac stress[2]. Newer emerging techniques, including navigation TKA and robotic surgery, aim to reduce the insult to the medullary canal of the distal femur and improve prosthetic alignment. Importantly, navigation TKA mitigates blood loss and the dissipation of systemic emboli associated with conventional TKA. Our previous publication showed that navigation TKA can lead to lower levels of surgery-stimulated endothelial injury markers as compared with conventional TKA[3].

Previous research into patients undergoing hip or knee surgery has found that post-surgical trauma-induced immune reactions affect the rates of postoperative recovery, infection and mortality[4–7]. We hypothesize that the reaming process in conventional TKA may incite a substantial inflammatory response, reflected by high levels of inflammatory markers in the serum and Hemovac drainage fluid.

## Materials and methods

### Patients

This prospective comparative study was approved by the Institutional Review Board of Kaohsiung Chang Gung Memorial Hospital (IRB 101-0050C) covering the period from 2013/01/16 to 2015/12/13. All study participants were recruited between 2013/02/08 and 2015/12/09.This study was registered in the ClinicalTrials.gov system (ID: NCT03163888). Failure by the local ethics committee to mandate the registration process before subject recruitment delayed the registration process. The study authors confirm that all ongoing and related trials for this intervention are registered. The protocol used for this trial and the accompanying TREND checklist are available as supporting information (see S1 Checklist and S1 Protocol).

The study design is similar to our previous work [3]. Patients requiring TKA for advanced knee OA presented to the outpatient department and were scheduled for TKA surgery. When the patients presented to the clinic, they were permitted to choose their preferred surgeon. Patients who consulted study author CJW underwent conventional TKA; those visiting study author JYK underwent navigation TKA. Both senior authors had performed more than 1,000 TKAs using their familiar method respectively before our study. Throughout the study period, neither specialist swapped to the alternative method in order to avoid performance bias and prolongation of operation time. At admission, the patients did not know which TKA method had been allocated to either surgeon. After admission and preoperatively, all patients were asked whether they were willing to participate in this study. The patients knew their allocation when they agreed to participate in the study and completed written informed consent. No patient shifted to the other group during the study. Those with autoimmune diseases, malignancies, previous knee surgery or post-traumatic arthritis were excluded.

The minimum sample size required for each group was calculated because of the pilot nature of the study. The priori power calculation (G*Power 3.1.9.2 software: http://www.gpower.hhu.de/en.html) used a 2-tailed Wilcoxon signed-rank test to calculate the sample size of at least 27 for each group (calculated effect size: 0.8; α level: 0.05; power: 80%; allocation ratio: 1).We finally planned to enroll at least 40 patients for each group after referencing the pertinent publications[3, 8].

All TKA procedures were performed under general anesthesia. Any anticoagulation regimen was discontinued prior to surgery. Procedural differences between the navigation and conventional techniques are described below[3].

### Computer-assisted navigation TKA

Bone cuts were guided and mapped using infrared-based navigation systems (Vector Vision; Brain LAB, Heimstetten, Germany). Two reference arrays were fixed to the distal femur and proximal tibia and tracked by an infrared camera. The hip joint center, distal femur and proximal tibia articulating surfaces, as well as the medial and lateral malleolus of the ankle, were mapped and registered. The femoral component was referenced to the anterior cortex of the distal femur. The rotation of the femoral component was referenced upon the epicondylar line, Whiteside line, and posterior condylar line. The size and position of the femoral prosthesis was determined by the navigation system. The distal femur cut and chamfer cut were guided by the navigation system without reaming the medullary cavity.

An extramedullary guide determined the level of tibia cutting, the varus-valgus angle and tibia slope. The rotation of the tibia component was accommodated to fit the femoral component. An extramedullary guiding rod was used to reference the ankle joint center, assisting with the determination of the rotation of the tibial component. Bone cutting was achieved under real-time navigation.

After completing the bone cut, femoral and tibia components (LPS-Flex system, Nexgen; Zimmer, Warsaw, IN, USA) were implanted with antibiotic-loaded cement fixation. A neutral mechanical axis with a deviation of less than 1 degree was achieved after soft tissue balancing under real-time navigation. A 1/8-inch Hemovac (Zimmer Hemovac; Zimmer, Warsaw, IN, USA) was left in place for 24 hours.

### Conventional TKA

Femoral bone cuts (distal and chamfer cuts) were guided by intramedullary guiding instruments. The proper size and positioning of the prosthesis (LPS-Flex system, Nexgen; Zimmer, Warsaw, IN, USA) was based on the surgeon’s discretion, and the femoral and tibia components were implanted with antibiotic-loaded cement fixation. A 1/8-inch Hemovac (Zimmer Hemovac; Zimmer, Warsaw, IN, USA) was kept in place for 24 hours.

### ELISA assay

Five milliliters of Hemovac drainage fluid was processed to collect supernatants. Ten milliliters of venous blood drawn from each participant prior to the procedure and again at 24 and 72 hours after TKA was processed to collect sera and stored at –80°C until analysis. Concentrations of inflammation markers in sera and drainage supernatants were measured using ELISA kits (R & D Systems). The author (Ya-Hung Hsu) performing the ELISA assay was blinded to the patient profile and group allocation via de-identification and anonymization of individual patient data.

### Statistical analyses

The data are shown as median values with lower and upper quartiles, which were expressed as median (lower quartile, upper quartile). Categorical variables were compared by Chi-square testing. The Mann-Whitney U test compared differences between groups. The Friedman test was used for the repeated measures analysis of repeated within-group comparisons for continuous variables and the Wilcoxon signed-rank test was used for post hoc analysis. All statistics were performed by SPSS software, and a p value of < 0.05 was deemed to be statistically significant.

## Results

**Fig 1:**
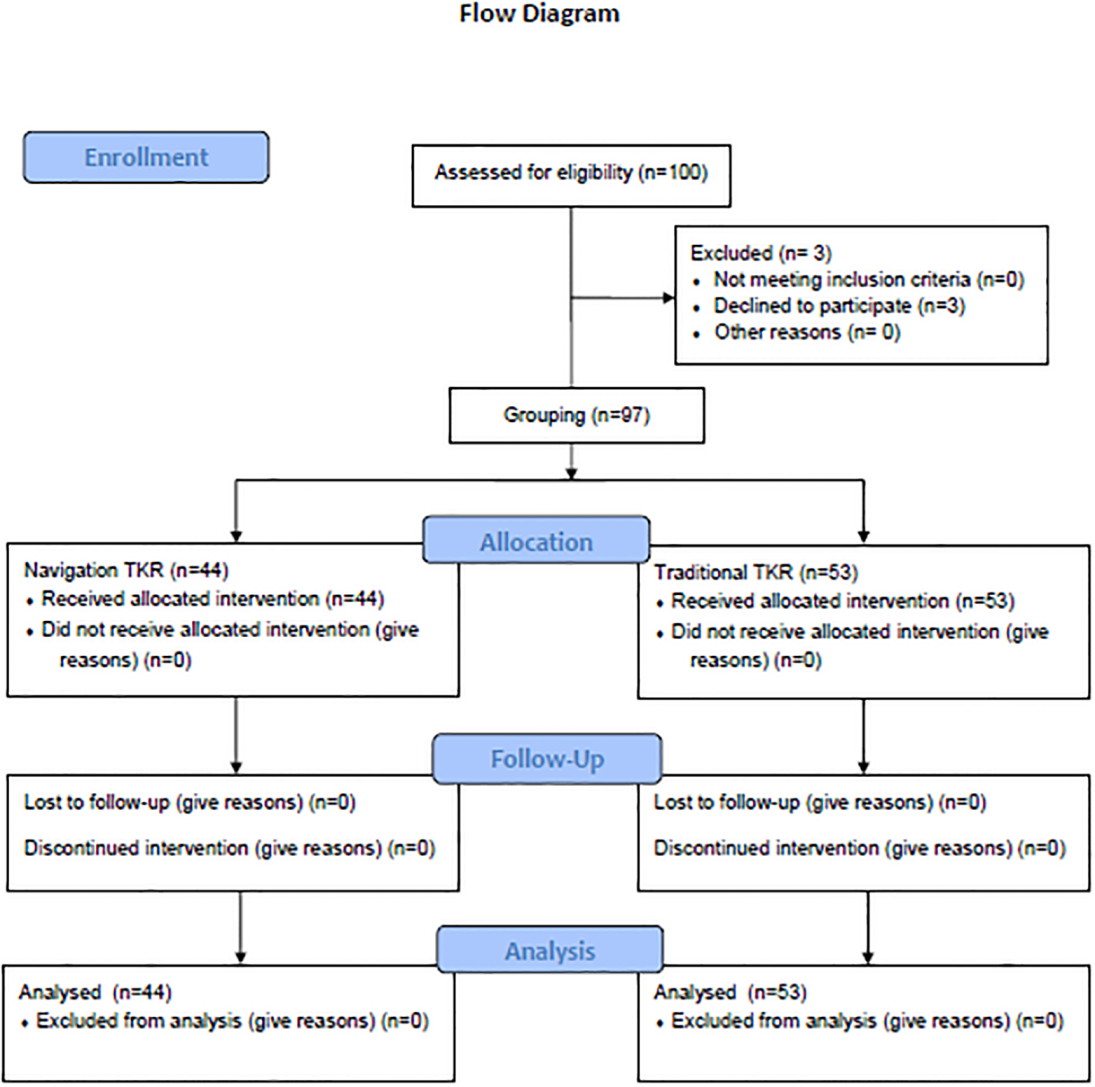
Flowchart of the participants at each stage of the study.

A total of 100 patients fulfilled the study inclusion criteria between February 2013 and December 2015; 46 patients were allocated to navigation TKA and 54 to traditional TKA. After excluding 3 patients who failed to provide valid written informed consent, 97 participants were enrolled; 44 underwent navigation TKA and 53 received conventional TKA. All 97 study participants completed the baseline and postoperative blood sampling and the collection of Hemovac drainage fluid (Fig 1).

There were no between-group differences in gender (p = 0.20), operated side (p = 0.61), age (p = 0.22), or body mass index(p = 0.23).A higher proportion of participants in the navigation group compared with those in the conventional group were diagnosed with coronary artery disease and equivalents (including diabetes, chronic kidney disease, carotid artery disease, peripheral arterial disease, and abdominal aortic aneurysm) (29.5% vs 13.2%,p = 0.047) (Table 1)[9].

**Table 1:** Baseline characteristics of patients undergoing conventional or computer-assisted navigation TKA.

	Conventional	Navigation	p-value
Gender			
Male	17 (32.1%)	9 (20.5%)	0.20
Female	36 (67.9%)	35 (79.5%)	
Operative side			
Left	19 (35.8%)	18 (40.9%)	0.61
Right	34 (64.2%)	26 (59.1%)	
Age (years)	70.6 ± 1.04	68.6 ± 1.20	0.22
BMI	28.5 ± 0.60	27.5 ± 0.59	0.23
Coronary artery disease & ^#^ equivalents	7 (13.2%)	13 (29.5%)	0.047

^#^ The equivalents included diabetes, chronic kidney disease, carotid artery disease, peripheral arterial disease, and abdominal aortic aneurysm.

The skin-to-skin closure time was 106 (88, 127) minutes in the conventional group and 102 (92~109) minutes in the navigation group (p = 0.17).

At baseline, serum levels were comparable between the two groups for IL-6 (p = 0.60), IL-10 (p = 0.17), TNF-α (p = 0.90) and TGF-β1 (p = 0.65).The Friedman p-values were all < 0.01 for IL-6, IL-10, TNF-α and TGF-β1 levels in the conventional group; corresponding values were < 0.01, 0.06, < 0.01 and < 0.01, respectively, in the navigation group. Thus, no significant intra-group difference was noted for serum IL-10 levels in the navigation group. The respective p-values of post hoc Wilcoxon signed-rank testing are shown in Tables 2–5. Generally, post-TKA levels of all 4 markers were higher than baseline in post hoc analyses.All exceptions were in the navigation group. TNF-α levels at 24 hours (p = 0.052) (Table 4) after TKA were comparable to baseline values, while TGF-β1 values at 24 hours (p < 0.01) and 72 hours (p <0.01) after TKA were all lower than baseline (Table 5).

**Table 2:** Serum IL-6 concentrations (pg/mL) prior to TKA, then at 24 and 72 hours after TKA.

	Navigation	Conventional	^a^ p-value
Baseline	1.02 (0.65, 2.79)	1.06 (0.64, 1.87)	0.60
24 hours after TKA	17.40 (6.33, 27.06) ^b^ p < 0.01	30.58 (27.42, 32.63) ^b^ p < 0.01	< 0.01
72 hours after TKA	13.08 (7.99, 23.29) ^c^ p < 0.01	18.53 (12.35, 30.85) ^c^ p < 0.01	< 0.01
24 hours—baseline	17.06 (4.25, 26.63)	29.39 (26.55, 31.32)	0.02
72 hours—baseline	12.27 (5.18, 21.74)	16.87 (12.14, 30.34)	0.01

^a^: p-value for between-group comparisons by Mann-Whitney U test.

^b^,^c^: Post hoc Wilcoxon signed-rank p-value for intragroup comparisons between values at 24 h (^b^) and 72 h (^c^) after TKA and preoperative baseline values. The respective Friedman p-values are described in the main text.

**Table 3:** Serum IL-10 concentrations (pg/mL) prior to TKA,then at 24 and 72 hours after TKA.

	Navigation	Conventional	^a^ p-value
Baseline	1.53 (0.70, 2.25)	1.91 (1.78, 2.11)	0.17
24 h after TKA	1.80 (1.34, 2.67)	2.47 (2.17, 3.35) ^b^ p < 0.01	0.02
72 h after TKA	1.09 (0.28, 2.09)	2.41 (2.11, 2.84) ^c^ p < 0.01	< 0.01
24 h—baseline	0.51 (–0.49, 1.57)	0.83 (0.36, 1.43)	0.16
72 h—baseline	–0.40 (-1.36, 0.74)	0.48 (0.12, 1.01)	< 0.01

^a^: p-value for between-group comparisons by Mann-Whitney U test.

^b^,^c^: Post hoc Wilcoxon signed-rank p-value for intragroup comparisons between values at 24 h (^b^) and 72 h (^c^) after TKA and preoperative baseline values. The respective Friedman p-values are described in the main text.

**Table 4:** Serum TNF-α concentrations (pg/mL) prior to TKA,then at 24 and 72 hours after TKA.

	Navigation	Conventional	^a^ p-value
Baseline	0.74 (0.51, 0.99)	0.66 (0.57, 1.01)	0.90
24 h after TKA	0.73 (0.46, 1.02) ^b^ p = 0.052	0.98 (0.71, 1.65) ^b^ p < 0.01	0.01
72 h after TKA	1.31 (1.04, 1.70) ^c^ p < 0.01	1.73 (1.25, 2.11) ^c^ p < 0.01	0.045
24 h—baseline	–0.04 (–0.41, 0.19)	0.36 (0.04, 0.89)	< 0.01
72 h—baseline	0.58 (0.04, 1.11)	0.98 (0.55, 1.39)	0.07

^a^: p-value for between-group comparisons by Mann-Whitney U test.

^b^,^c^: Posthoc Wilcoxon signed-rank p-value for intragroup comparisons between values at 24 h (^b^) and 72 hours (^c^) after TKA and preoperative baseline values. The respective Friedman p-values are described in the main text.

**Table 5:** Serum TGF-β1 concentrations (pg/mL) prior to TKA,then at 24 and 72 hours after TKA.

	Navigation	Conventional	^a^ p-value
Baseline	3024.71 (2983.46, 3048.13)	3030.32 (3002.55, 3059.34)	0.65
24 h after TKA	2980.81 (2941.78, 3024.04) ^b^ p < 0.01	3106.75 (3079.72, 3128.60) ^b^ p < 0.01	< 0.01
72 h after TKA	2974.60 (2940.81, 3003.29) ^c^ p < 0.01	3101.74 (3059.10, 3131.50) ^c^ p < 0.01	< 0.01
24 hours—baseline	–48.18 (–82.94, –12.37)	63.24 (43.96, 100.39)	< 0.01
72 hours—baseline	–55.16 (–87.23, –11.23)	63.71 (38.79, 137.51)	< 0.01

^a^: p-value for between-group comparisons by Mann-Whitney U test.

^b^,^c^: Post hoc Wilcoxon signed-rank p-value for intragroup comparisons between values at 24 h (^b^) and 72 h (^c^) after TKA and preoperative baseline values. The respective Friedman p-values are described in the main text.

At 24 hours after surgery,serum levels of IL-6 (Table 2), IL-10 (Table 3), TNF-α (Table 4) and TGF-β1 (Table 5) in the navigation group were 43.1% (p < 0.01), 27.1% (p = 0.02), 25.5% (p = 0.01) and 4.1% (p< 0.01) lower than those in the conventional group.Similarly, at 72 hours after TKA, concentrations of IL-6 (Table 2), IL-10 (Table 3), TNF-α (Table 4) and TGF-β1 (Table 5) in the navigation group were 29.4% (p = 0.02), 54.8% (p < 0.01), 24.3% (p = 0.045) and 4.1% (p < 0.01) lower than those in the conventional group.

At 24 hours, postoperative increments in serum IL-6 (p < 0.01) (Table 2), TNF-α (p < 0.01) (Table 4) and TGF-β1 (p< 0.01) (Table 5) were significantly lower in the navigation group versus those in the conventional group. This trend continued at 72 hours, with significantly higher increases from baseline observed in serum IL-6 (p = 0.01) (Table 2), IL-10 (p<0.01) (Table 3), and TGF-β1 (p < 0.01) (Table 5) in the conventional group versus those in the navigation group.

The Il-10 levels in Hemovac drainage fluid collected 24 hours after operation were significantly lower (p <0.01) in the navigation group than in the conventional group (Table 6).

**Table 6:** Concentrations of inflammatory markers in Hemovac drainage fluid fromeach TKA cohort.

	Navigation	Conventional	p-value
IL-6	12.32 (12.17, 12.60)	12.28 (12.11, 12.55)	0.48
IL-10	8.55 (4.93, 9.37)	12.32 (8.36, 15.95)	< 0.01
TNF-*α*	1.70 (1.18, 2.28)	1.37 (0.79, 2.31)	0.37
TGF-*β*1	3021.79 (2987.00, 3066.53)	3023.63 (2979.14, 3053.73)	0.46

All study participants were followed for 6 months after TKA for any complications. One patient from the conventional group visited the urologist’s clinic due to dysuric symptoms 2 months after surgery, and one patient from the navigation group visited the otolaryngologist’s clinic due to tinnitus symptoms 3 months after TKA. No other major complications were noted in either group.

## Discussion

In this study, we observed smaller increases from baseline in postoperative serum IL-6, IL-10, TNF-α and TGF-β1 levels among patients in the navigation TKA cohort compared with those in the conventional TKA group. IL-10 concentrations in Hemovac drainage fluid collected 24 hours after TKA were also lower in the navigation group. The between-group differences in local and systemic inflammation after navigation and traditional TKA have not previously been reported and deserve attention.

A nationwide study from Denmark demonstrates an increased incidence of AMI after TKA[2]. The study authors showed that the AMI risk increases 30.9-fold during the first 2 weeks after TKA. However, the absolute 6-week AMI risk after TKA was only 0.21%. This low absolute risk means that any study seeking to validate the benefits of a reaming-free procedure in terms of AMI, or other major morbidity or mortality, requires an extremely huge sample size. Recent studies have failed to show superiority for the navigation technique over conventional TKA in terms of clinical results, image findings and survival rates [10, 11]. Some researchers have also questioned the role of sealing the femoral canal in mitigating blood loss [12]. However, none of these “high quality” studies are sufficiently powered to conclusively demonstrate or refute whether a real difference exists between the two techniques as to risk of mortality and major morbidity. We have therefore attempted to demonstrate the risk reduction effect of the reaming-free navigation technique by comparing surrogate endpoints, such as biomarkers with prognostic value for major complications. Our previous work demonstrated a smaller postoperative increase in the navigation group in serum levels of cell adhesion molecules, which reportedly reflect the severity of ischemic cadiomyopathy[3]. For this study, inflammation markers were selected according to evidence in the previous publications. All of the selected markers have prognostic value for mortality, major morbidity and functional recovery after lower limb surgery.

Sun et al. recruited 127 elderly hip fracture patients and 60 elderly volunteers for comparison [13]. Their study showed that serum levels of TNF-α, IL-6 and IL-10 were all higher among the fracture patients. TNF-α levels > 55.27 pg/mL and IL-6 levels > 79.50 pg/mL on post-operative day 1 were independent predictors for mortality at 6 months, while IL-6 levels > 80.50 pg/mL and IL-10 levels > 80.50 pg/mL on postoperative day1 were independent predictors for mortality at 1 year. Among the survivors, IL-6 levels > 83.15 pg/mL on day 1 predicted subsequent major complications, such as pneumonia. Beloosesky et al. examined 41 hip fracture patients and found that serum TGF-β1 levels among patients with infection or cardiovascular events were significantly higher than in patients without such complications (p = 0.039). The authors assumed that the increase in TGF-β1 among patients with complications is due to its anti-inflammatory effects in response to increased pro-inflammatory cytokines associated with complications[14]. The two studies supported the value of inflammatory markers in predicting major complications after hip fracture surgeries.

Inflammatory responses also correlate with functional recovery after total hip arthroplasty (THA) [15]. Hall et al. examined 102 patients undergoing THA and found that serum IL-6 levels at 24 hours after THA correlated with the recovery time to walk for 10 and 25 minutes. The extent of inflammation also correlated with functional recovery after TKA [8]. Langkilde et al. recruited 60 patients undergoing TKA and found that at 26 weeks after surgery, IL-10 levels were negatively associated with the gain in walking distance. The authors attributed the elevation in IL-10 to surgery-induced damage response. These studies attest to the value of inflammatory markers in predicting functional recovery after TKA and THA.

Based upon the publications mentioned above, inflammation markers harbor prognostic potential for major complications and functional recovery after lower limb surgery. Levels of these markers are higher among patients undergoing conventional TKA compared with those undergoing navigation TKA. Moreover, serum TGF-β1 levels increased after conventional TKA but decreased after navigation TKA. Serum TGF-β1 levels reflect the severity of OA [16–18]. We consider that the lowering of TGF-β1 levels after removal of severe arthritic tissue exceeded the increase in TGF-β1 induced by surgical trauma, leading to an overall decline in TGF-β1 after navigation TKA. Similarly, IL-10 is considered to reflect the extent of arthritic severity and the extent of surgery-induced trauma [8, 19]. Comparable serum IL-10 levels seen before and after navigation TKA may be due to a balanced effect between removal of severe arthritic tissue and surgery-induced trauma.

There are limitations to our study. Firstly, we cannot demonstrate superiority of navigation over conventional TKA in terms of functional recovery or major complications due to the small sample size. Secondly, the levels of inflammation markers in both groups were all substantially lower than the values that reportedly predict mortality and morbidity[13]. However, the timing of our observation overlapped exactly with the “hazardous first two weeks” for AMI after TKA [2]. In the conventional group, serum IL-6 levels rose 28.8-fold from baseline by 24 hours after TKA, while serum TNF-α levels increased persistently from baseline to 72 hours after TKA. Although the serum inflammatory markers failed to reach the values that predict mortality, the substantial and persistent increase within the “hazardous first two weeks” after conventional TKA deserves emphasis. Thirdly, the study participants were allowed to choose whichever surgeon they preferred, which therefore jeopardized the randomization process despite the stringent de-identification and anonymization process. However, the patients did not know which technique would be performed until admission, and no patient shifted to the alternative group during the study. Demographic profiles were similar between the groups, as were the skin-to-skin closure times. The strategies mentioned above may mitigate the impact of potential selection bias [3].

## Conclusions

Conventional TKAs lead to higher level of inflammation markers in serum and hemovac drainage. This study highlights low systemic and local inflammation markers as emerging biochemical indicators that augment the merits of navigation TKA. The correlation between the extent of inflammation and the functional recovery or even major complication deserves further investigation.

## Supporting information

S1 Checklist TREND statement checklist for the current study. (PDF)

S1 Protocol The original document of our study protocol(in Chinese). (DOCX)
